# Optical Blaster: Launching Nanostructured Microrockets out of an Optical Trap by a Single Laser Beam

**DOI:** 10.1021/acsnano.5c07197

**Published:** 2025-07-18

**Authors:** Yera Ussembayev, Yuki Arakawa, Filip Beunis, Anne B. Spoelstra, Tom Bus, Albert P. H. J. Schenning, Kristiaan Neyts

**Affiliations:** † LCP Research Group, Ghent University, Technologiepark 126, Gent 9052, Belgium; ‡ Center for Nano and Biophotonics, Ghent University, Technologiepark 126, Gent 9052, Belgium; § SFD Research Group, Eindhoven University of Technology,Eindhoven 5600 MB, The Netherlands; ∥ Department of Applied Chemistry and Life Science, Toyohashi University of Technology, Toyohashi 441-8580, Japan; ⊥ CMEM Research Group, Eindhoven University of Technology, Eindhoven 5600 MB, The Netherlands; # 58207 Hong Kong University of Science and Technology, Clear Water Bay, Kowloon 000000, Hong Kong, China

**Keywords:** optical trapping and manipulation, particles, liquid crystals, chirality, photonic bandgap

## Abstract

Precise manipulation of microscopic objects is crucial for applications in biomedicine, robotics, and nanotechnology. However, achieving stable trapping, controlled release, and rapid propulsion with a single laser beam remains a significant challenge. Here, we elucidate the optical forces and torques exerted on chiral liquid crystal polymer microparticles when the laser wavelength is within their photonic bandgap and demonstrate the application in an optical blaster. Photons with polarization handedness opposite that of the chiral helical structure in the particle are transmitted and can be used to trap the particle, while photons with the same polarization handedness lead to a strong recoil effect. By leveraging this mechanism, we create an optical blaster that first loads the microparticles into the optical trap and subsequently launches them as microrockets by switching the circular polarization handedness of the light beam. The particles achieve propulsion speeds up to 234 μm s^–1^ and finally levitate well above the laser focus after a balance between gravity and the optical force is reached. The optical blaster concept holds promise for diverse applications in ultrafast cargo transport, soft microrobots, microsurgery, and the advanced nanomanipulation of small objects.

## Graphical abstract

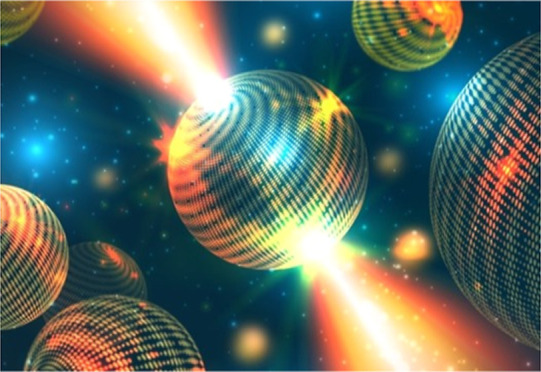

## Introduction

Optical manipulation has emerged as a key research field in optics and photonics, driving advancements in colloidal and biological sciences, where precise control of microscopic objects is essential. However, effectively using a single laser beam for both optical trapping and light-powered propulsion remains a major challenge. Mastering this capability would enable precise lateral positioning of optically trapped objects and extend the axial range, which is typically constrained by tight focusing of the laser beam. Nanostructured materials, including metasurfaces^1^ (MSs) and cholesteric liquid crystals^2^ (LCs), offer a promising approach to overcome these limitations due to their photonic bandgap (PBG), which allows selective reflection, transmission, or confinement of light at specific wavelengths.^3^ In particular, the PBG can be tailored to enhance the radiation pressure acting on small objects, enabling dynamic control of their motion. For instance, such materials have been explored for light-powered transport systems, including metavehicles,^4^ microdrones,^5^ plasmonic nanomotors,^6,7^ and even light-sailing spacecraft.^8,9^ However, the strong radiation pressure usually prevents stable trapping in a highly focused laser beam when its wavelength falls within the PBG spectral range of the material.^10,11^ To address this, nanostructured chiral metasurfaces^1,12,13^ (CMSs) and chiral nematic (or cholesteric) liquid crystals^2,14^ (CLCs) offer promising opportunities as their chirality induces very different interactions for left- (LCP) and right-handed circular polarized light^15–17^ (RCP) and excellent control over optical forces and torques.^18–20^ Compared to CMSs, CLC materials present key advantages, including minimal optical losses,^2,21^ tunable optical properties, and programmable helical self-assembly,^22,23^ making them ideal for optical trapping applications. The ability to optically trap objects with chiral PBGs would enable extended control over light-driven micro- and nanovehicles and significantly expand their potential applications across various fields, including single-molecule biophysics,^24^ miniaturized robotic systems,^25^ and micro- and nanofluidics.^26^ Moreover, optical trapping of PBG materials will offer new insights into undiscovered optomechanical phenomena at the micro- and nanoscale, potentially enhancing and transforming optical manipulation techniques.

To date, several studies have reported on the optical trapping of liquid crystal particles and droplets.^21,27–29^ Many studies focus on micro-objects containing nematic liquid crystals,^21,27^ which lack chirality and show bidirectional rotation for both circular polarization handedness due to anisotropy.^30,31^ Nevertheless, there has been some research on the optical trapping of chiral liquid crystal microparticles with PBGs outside the spectral range of a trapping laser.^20,32,33^ Recent reports^34–36^ have demonstrated the synthesis of CLC polymer microparticles with complex nanostructures based on molecular self-organization.^22^ These cholesteric LC beads are largely composed of a helical stack of planar layers^36^ or a concentric (onion-like) arrangement^34,35^ of LC. When applied for optical tweezers, these particles exhibit a unique spinning behavior for different circular polarizations due to their chirality.^32,33^ Particularly, the particles with a parallel stacking of CLC layers demonstrate uni- or bidirectional rotation depending on the alignment of the particle within the optical trap.^32^ Onion-structured CLC particles, on the other hand, show only unidirectional rotation due to the rotational symmetry of the particles.^33^ In addition, optical trapping of particles with concentric CLC layers is feasible with light that matches the chiral PBGs if the polarization has the opposite handedness.^18^ This selective interaction enables stable trapping by exploiting the polarization-dependent transmission and reflection properties of the chiral photonic structures. These unique optomechanical properties endow CLC particles with significant potential for various applications, including helicity-dependent optical transport^37^ and sorting,^26,38^ rotary micro- and nanomotors,^32,33^ and spinning sensors.^21,32^

Here, we expand the optical trapping technique by increasing the axial manipulation range and using custom-designed chiral LC microparticles with a PBG that matches the wavelength of the trapping laser. This research presents intriguing scientific possibilities that have not been observed in nonchiral materials with a PBG. Notably, the chirality in CLC materials causes the PBG to become polarization-dependent, implying that light with LCP and RCP exerts completely different optical forces and torques. We can leverage this effect to create an “optical blaster”. Complementing other tools in the optical manipulation toolbox (e.g., optical tweezers,^24^ optical torque wrench,^39^ optical gun,^40–42^ and optical tractor^43^), this device is capable of holding and launching CLC “microrockets” by switching the circular polarization from one (holding) to the opposite (launching) handedness, thereby acting as a trigger. The ability of the optical blaster to switch between trapping and launching states with high precision and fast response will make it a versatile tool that can be used in many disciplines, ranging from physics and engineering to biology and medicine. Moreover, this novel concept together with CLC microrockets may reveal unprecedented optomechanical phenomena at the micro- and nanoscale. Hence, our research aims to control the optical forces and torques exerted on CLC microparticles by altering the circular polarization state of the trapping laser, paving the way for advanced manipulation techniques in optical trapping.

## Results and Discussion

In this work, we investigate how the interplay between the photonic bandgap and chirality influences the optical trapping of nanostructured materials and how the resulting optical forces and torques can be applied in this optical manipulation technique. To address these questions, we first fabricate two different types of right-handed chiral liquid crystal polymer microparticles (Figure 1) by using suspension photopolymerization^32,35,36^ (Methods). Figure 1a shows a cross-sectional transmission electron microscopy (TEM) image of the produced CLC microparticles, where the liquid crystal director rotates periodically throughout the particle due to chirality, forming a helical structure with a planar stacking (Figure 1c, top row) of layers with different refractive indices.^36^ Another type of the fabricated particles has CLC layers arranged concentrically in an onion-like structure^35^ (Figure 1b) with the helical axis oriented radially, except for a region containing a radial defect line^35^ (aligned in the +*z*-axis in Figure 1c, bottom row). One period in the pattern determines half of the helical pitch of the CLC structure. For example, Figure 1a,b shows CLC microparticles with chiral pitch sizes *p* of about 600 and 620 nm, respectively. Assuming an average refractive index *n̅* = 1.6, the corresponding central PBG wavelengths for normally incident light $\lambda_{PBG}=\mathrm{n̅}p$ are approximately 960 and 990 nm. Both types of particles have a regular spherical shape with a diameter *d*_p_ of 7 ± 5 μm. Inspired by the observed CLC layer structures, we developed an analytical model^32^ (Methods) to describe the director configuration *L* for planar (Figure 1c, top row) and concentric (Figure 1c, bottom row) layers. This analytical framework for the CLC director configuration serves as a foundation for modeling optical trapping forces and torques.

**Figure 1:**
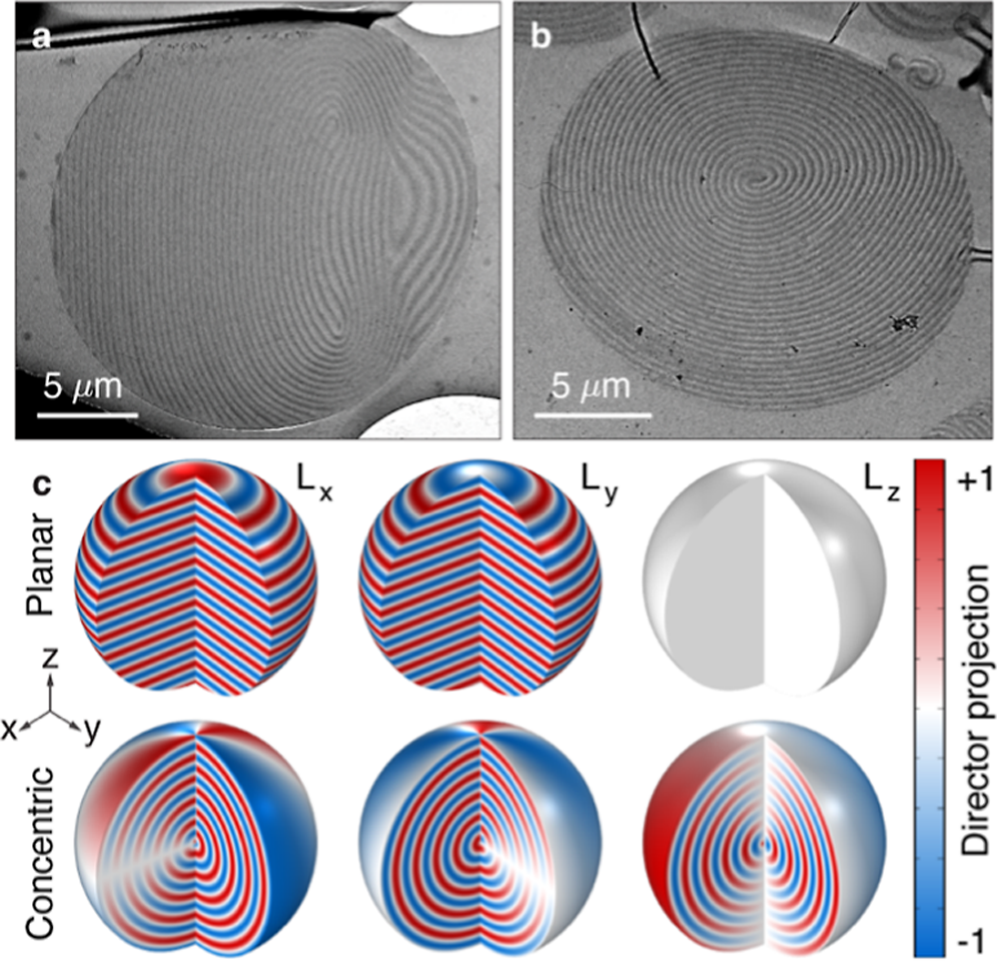
Internal nanostructure of the designed chiral liquid crystalline microparticles. (a) Transmission electron microscopy (TEM) image of the cross-sectional view of a CLC microparticle with the planar stacking of the helical liquid crystal layers with a helical pitch of 600 nm. (b) TEM micrograph of a concentric (onion-like) arrangement of the helical liquid crystal layers with a chiral pitch size of 620 nm. (c) Spatial variation of the projection of the director *L* on the three principle axes (*L*_*x*_—left column, *L*_*y*_—middle column, *L*_*z*_—right column) for the planar (top row) and concentric (bottom row) layers of CLC microparticles. The diameter of the particle is 6 μm and the size of the helical pitch is 600 nm, which forms a photonic bandgap in the near-infrared wavelength range. The axis system shown here and further is right-handed.

Next, we numerically calculate (Methods) the optical forces and torques exerted by a strongly focused Gaussian beam on microparticles with different layered CLC structures as shown in Figure 1c. The modeled microparticles have a diameter of 2 μm, right-handed chirality, and a helical pitch of 600 nm, forming a NIR photonic bandgap that includes the trapping laser wavelength (λ_0_ = 975 nm). We consider particles with the planar stacking of CLC layers aligned with their helical axis parallel to the propagation direction, while onion-like CLC particles are oriented with the disclination line perpendicular to the wavevector. The results of these calculations are displayed in Figure 2, where the generated optical force is shown as a function of the particle’s axial position (bottom graphs) within the laser beam, as depicted in the illustrations at the top. In general, optical trapping relies on the balance between two optical forces: the gradient force due to the strong focusing of the beam and the scattering force generated by the radiation pressure. Stable trapping is achieved when the gradient force in the focus of the beam is larger than the scattering force and is able to compensate thermal diffusion. When the CLC particle is illuminated with LCP (Figure 2a), gradient and scattering forces compensate each other in the equilibrium position, where the particle remains trapped. In contrast, for RCP (Figure 2b), the optical scattering force always exceeds the gradient force, pushing the particle away from the focus and rendering it untrappable. The calculated pushing forces exerted on particles with the planar stacking of CLCs are almost five times stronger than those experienced on onion-like CLC particles. The orientation of the CLC particle within the laser focus also influences whether the particle can be optically trapped under LCP (Extended data Figure S1a). For instance, when a particle with planar stacking is aligned with its helical axis perpendicular to the laser propagation direction, it becomes untrappable. For onion-structured particles, this effect is observed only when the particle is aligned with its disclination line parallel to the light propagation direction. We also calculated the optical torques acting on the CLC particles for different circular polarizations (Extended data Figure S1b), revealing another chirality-dependent behavior. Notably, particles with planar CLC layers experience strong torque under RCP and negligible torque, nearly zero, under LCP. However, for particles with concentric CLC layers, both circular polarizations generate optical torques due to the defect line aligned perpendicularly to the light propagation direction, acting as a lever. Additionally, we examined how optical forces are modified when the chiral pitch is either 400 nm (PBG in the red wavelength range) or 250 nm (PBG in the blue wavelength range), as exemplified in Extended data Figure S2. When the PBG shifts away from the trapping laser wavelength, the chirality dependence of the optical forces observed for *p* = 600 nm disappears.^32^ This indicates that the interaction between the laser beam and the CLC structure is highly dependent on the correspondence between the PBG and the laser wavelength. For this experiment, the difference between the refractive indices *n*_e_–*n*_0_ should be sufficiently large to obtain a wide bandgap and strong reflectivity for different angles of incidence for RCP. Overall, the obtained numerical results confirm our hypothesis that the handedness of circular polarization exerts different optical trapping forces and torques on CLC microparticles when their PBGs match the trapping wavelength.

**Figure 2:**
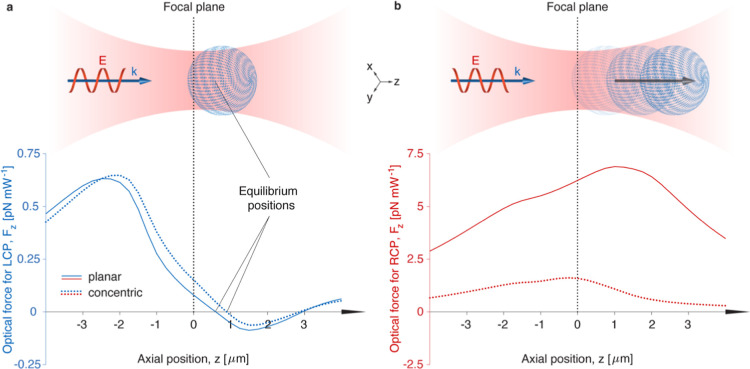
Numerical calculation of the optical forces on CLC microparticles exerted by highly focused Gaussian beams. Calculated optical forces as a function of the axial position for LCP (a) and RCP (b) and light propagating in the *z*-direction and illuminating the particles as depicted in the schematic images at the top. The illustrations are not to scale and the vertical axes are different. For LCP (a), there are equilibrium positions, which indicate stable optical trapping, whereas for RCP (b), there is a strong optical scattering force that pushes the particles away from the laser focus. The diameter of the calculated microparticle is 2 μm and its helical pitch size is 600 nm. The trapping laser wavelength is 975 nm.

The theoretically demonstrated dependence of optical forces on the handedness of circular polarization makes CLC microparticles promising candidates for creating an optical blaster. Our proposed idea involves switching the circular polarization from left-handed (trapping state) to right-handed (launching state), enabling this device to first hold CLC microparticles near the focus of the optical trap and then shoot them out, using light polarization as an effective trigger mechanism. To verify this concept experimentally, we employ optical tweezers^32,44^ (Methods). In this setup, the expanded NIR laser beam can be switched between left- and right-circular polarization by adjusting the AC voltage across the electrodes of a cell filled with nematic LC, placed before a static quarter-wave plate (QWP), as schematically shown in Extended data Figure S3. The switchable LC half wave plate can thereby invert the circular polarization handedness with a response time of 8 ms. Figure 3a presents a sequence of video frames showing a microparticle with concentric CLC layers when the circular polarization of the trapping laser is switched (Video file 1). Under LCP (blue frames), the particle remains stably trapped in the laser beam, as evidenced by the movement of another particle in the background during the first second, due to the motion of the stage. Under RCP (red frames), the trapped particle is launched like a rocket and propelled away from the focal plane within the next two seconds, as indicated by the changing diffraction pattern around the particle. The same behavior is observed for the microparticles with the planar stacking of CLCs (Video file 2). By analyzing the diffraction pattern of the particles (Methods), we could estimate the axial position as a function of time for three different particles with concentric CLC layers and one particle with the planar stacking of CLC layers (Figure 3b). The traces are aligned with their launching time at 1.2 s; before this point, the particles remain trapped under LCP, and after this point, they are launched under RCP. The shooting speed of the particle increases with particle size, as indicated by the increasing slope steepness of the linear fit to the data points shortly after the triggering moment. Overall, the experimental results in Figure 3 confirm our hypothesis and theoretical predictions (Figure 2).

**Figure 3:**
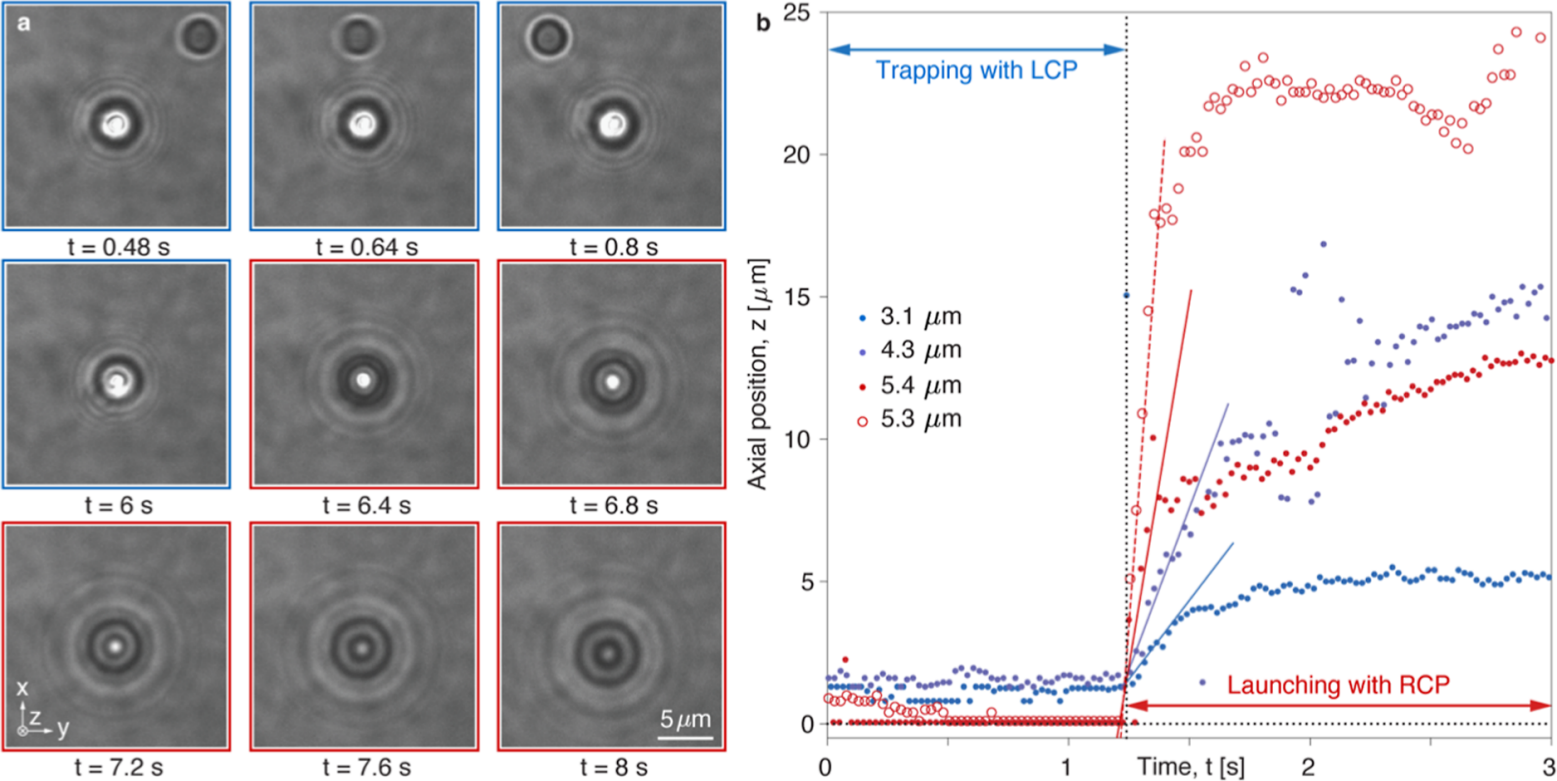
Experimental optical trapping and launching of CLC microparticles. (a) Nine sequential video frames of a particle with a diameter of 4.3 μm and a concentric arrangement of CLC layers, showing stable optical trapping with LCP (blue frames) and fast optical launching, triggered by RCP (red frames) at 6.2 s. (b) Position traces in the *z*-direction for four different microparticles (full dots for concentric and open circles for the planar stacking of CLC layers). The particles are optically trapped with LCP (blue double arrow indicates the trapping duration) and propelling through the medium with RCP (red double arrow indicates the propulsion duration). All traces are aligned with their launching time at 1.2 s, indicated by the black dotted line. The color lines show the linear fit to the initial measured data points, used to extract the particle velocity (solid lines for concentric arrangement and dashed lines for the parallel stacking of CLC layers).

The developed optical blaster based on the trapping and launching of CLC microrockets encompasses many interesting, previously unexplored, optomechanical effects. For instance, the CLC nanostructure inside the particles governs the trapping stability under LCP. The onion-shaped microparticles always exhibit consistent optical trapping. However, the particles with the planar stacking of CLC layers do not always maintain a trappable orientation, after a second trapping attempt is made after releasing the particle (Video file 3). Specifically, Figure 4a shows a sequence of video frames where this type of particles is optically trapped with LCP for the first four seconds (i) and then launched with RCP over the next four seconds. After the laser has been switched off (black frames) and the particle has dropped down to the glass surface due to gravity, we attempt to trap it again with LCP at *t* = 13 s (ii) and *t* = 17 s (iii). However, in both cases, the particle is scattered out of the laser focus, and it is only at *t* = 21 s (iv) that the particle is stably trapped again. According to our theoretical calculations (Extended data Figure S1a), this behavior originates from the alignment of the particle within the laser beam, where attempts (i) and (iv) illustrate successful trapping when the particle’s helical axis is more or less parallel to the propagation direction. In contrast, attempts (ii) and (iii) demonstrate the challenges associated with realigning the particle to a trappable orientation when the helical axis is, for example, oriented perpendicular to the propagation direction. This behavior is more common in particles larger than the average size, where an increased rotational drag can counteract the optical torque needed to reorient the particle into a trappable configuration. In contrast, smaller particles consistently adopt a favorable orientation, resulting in stable and repeatable trapping. Another important property of our CLC microparticles is their attainable optical torque and rotation. Both particle types with sizes below 5 μm slowly spin clockwise under LCP, as observed in Video file 1 with concentric CLC layers and Video file 2 with the planar stacking of CLC layers, with particle sizes of 4.3 and 4.4 μm, respectively. However, our theoretical predictions (Extended data Figure S1b) indicate that beads with the planar stacking of CLCs should experience nearly zero torque when trapped under LCP. This discrepancy occurs from particle to particle irregularly and may arise either from the structural irregularities of CLCs inside the particles or from the particles’ alignment within the optical trap, where they align their helical axis at a slight deviation angle from the beam propagation direction, acting as a lever for optical torque generation. For particles with a larger diameter (>5 μm), the exerted optical torque is insufficient to overcome the rotational drag experienced by the large beads (Video files 3, 5, and 6). Interestingly, when the particle size is smaller than ∼1.5 μm, there are fewer than three layers of chiral LC layers inside the bead, which is insufficient to form a well-pronounced PBG. Consequently, the scattering force under RCP does not launch the particle (Video file 4) but causes only a small axial displacement in the optical trap. Additionally, we observed light-induced levitation of the CLC microrockets, well above the focal plane of the trapping laser (up to ∼25 μm in Figure 3b) after the launch. For instance, Figure 4b shows time-lapse images (Video file 5) in which a particle is trapped (*t* = 1 s), launched (*t* = 2 s), and drops down under gravity when the laser is turned off (*t* = 8 s). However, when we keep the laser on (Video file 6), the same particle remains levitated above the laser focus for an extended period, as shown in Figure 4c. This effect arises due to diffraction of the laser beam after the focus point, which reduces the scattering force and the thrust of the microrocket with increasing *z*-values. At a certain height, the magnitude of optical and gravitational forces is the same, and they counterbalance each other, resulting in particle levitation. Finally, we also noticed an intriguing effect when a couple of attached particles with similar sizes (Figure 4d) are loaded (*t* = 6 s) and launched (*t* = 8.5 s) out of the laser focus (Video file 7). Apparently, the forces and torques during the launch are sufficient to separate the particles because after arriving at the bottom glass surface (*t* = 28.5 s), they are clearly separated. The exact mechanism of this process is unclear and is required for further investigation.

**Figure 4:**
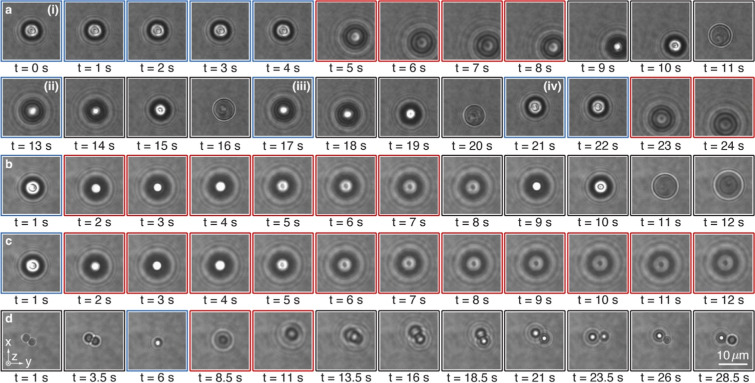
Time-lapse images of different experimental observations of CLC microrockets. Optical trapping and launching of a microparticle with a planar stacking of CLC layers (a), showing how the trapping of the bead depends on its alignment within the laser beam. Optical levitation of a particle with concentric layers of CLC layers when the laser light is turned off (b) and remains on (c). Optical separation of a couple of particles (d) after launching them out of the optical trap. The blue, red, and black frames correspond to left circular polarization, right circular polarization, and no laser, respectively.

To highlight the contrast between microrockets with planar stacking and concentric CLC layers and their attainable shooting speeds and forces, we summarize the experimental results in Figure 5. This diagram depicts the relationship between the particle diameter and the estimated forces along with an inset graph showing particle diameter versus launching speed. The analysis is based on 38 different microparticles, comprising 22 beads with concentric CLC layers and 16 particles with the planar stacking of CLC layers, that were trapped and launched. The main plot illustrates the different forces acting on the particles, including gravitational force *F*_grav_, drag force *F*_drag_, and optical force *F*_opt_ (Methods). The gravitational force is estimated according to Archimedes’ principle and increases with the cube of the diameter. The drag force is estimated from the speed, using Stokes’ law. The optical force is the sum of the magnitudes of the two forces according to Newton’s third law of motion because the acceleration force is negligible. In general, both optical forces and velocities increase with particle size due to the increasing number of CLC layers contributing to the light reflection. However, these parameters differ significantly between the two types of microparticles. This difference arises from the layered architecture of CLCs within the particles. For planar layers, incident photons recoil in the same backward direction as they would on a planar reflective surface. In contrast, for concentric layers, photons are reflected in various directions, similar to that for a convex mirror. Consequently, the total radiation pressure is greater for the planar stacking surface than for the concentric one.^8,9^ The measurements indicate that the attained optical force and speed are almost five times higher for microrockets with a planar stacking of CLCs compared to those with an onion-like structure, consistent with the calculated optical forces (Figure 2b) exerted under RCP (also about 5 times). As a result, the microrockets with a planar stacking of CLC layers achieve shooting speeds of up to 234 μm s^–1^ (inset graph in Figure 5), a record that to our knowledge has never been reported among light-powered linear micro- and nanovehicles in liquids. Accurate comparison of the calculated and measured optical forces is complicated due to losses in the high-NA objective lens, which may result in up to 70% power loss.^45^ Another difficulty is that the calculated force reaches its maximum within 2 μm above the focal plane and diminishes substantially away from the focus. Our camera measures speeds at a frequency of 40 Hz, making it difficult to resolve the traveled distance of a few micrometers with this frame rate. Nevertheless, the obtained theoretical and experimental results are in good agreement.

**Figure 5:**
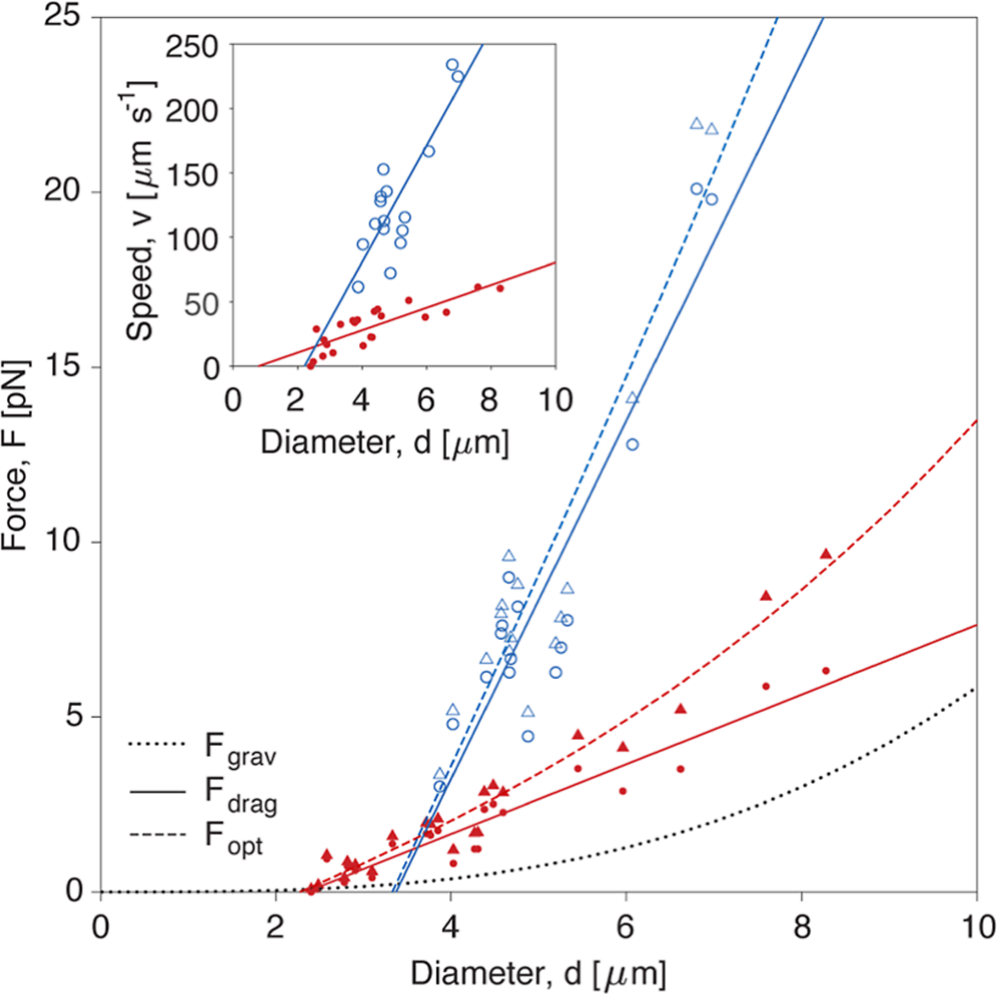
Experimentally estimated forces and speeds of CLC microrockets. The graph summarizes the dependency of different forces acting on CLC microparticles on their diameter under RCP. The red markers and solid lines represent measured data and linear fits for particles with a concentric internal structure, respectively, while blue symbols and solid lines indicate data and fits for beads with the planar stacking of CLC layers, respectively. Circles and triangles correspond to the measured drag and the estimated optical force, respectively. The black dotted line shows the calculated gravitational force as a function of the measured particle size. The dashed lines show optical forces as the sum of a linear fit to the drag forces and the calculated gravitational forces. The inset graph exemplifies the measured speed (markers) and linear fits (solid lines) depending on the diameter for different particles (red for onion-like structures and blue for planar stack of CLC layers).

## Conclusion

In conclusion, our developed optical blaster concept provides a significant advancement in understanding the optomechanical behavior of chiral liquid crystal microparticles with photonic bandgaps that match the trapping laser wavelength. Our experiments confirm the theoretical prediction that the handedness of circular polarization of the trapping beam has a profound impact on the optical force and torque experienced by CLC microparticles. Specifically, we demonstrated that LCP can stably trap these particles, while RCP propels them away, effectively turning the particles into microrockets driven by optical thrust. This phenomenon is attributed to the differential interaction of the chiral photonic bandgap with circular polarization of opposite handedness, leading to a selective enhancement of the optical scattering force under RCP illumination. The observed difference in the trapping and propulsion behavior between planar and concentric CLC microparticles further underscores the critical role of the internal nanostructure in dictating the optomechanical response. Planar CLC particles exhibit alignment-dependent trapping stability, with successful trapping occurring when the particle’s helical axis aligns parallel to the propagation direction of the laser beam as expected according to our theoretical results. In contrast, concentric CLC particles consistently achieve stable trapping, irrespective of their orientation, demonstrating their robustness for optical manipulation applications. This is illustrated in Video file 8, where we “juggle” three such beads by loading and shooting them out of the optical trap for two juggling rounds. The developed optical blaster is capable of switching between trapping the particle near the focus of the laser beam and launching it with high speed and a short response time, providing a versatile tool for micro- and nanoscale manipulation with extended axial control. A possible further modification is the use of a vortex beam instead of a Gaussian beam to achieve a larger torque for the same beam power. These innovative concepts not only enhance our ability to manipulate matter at the micro- and nanoscale, enabling the assembly and disassembly of aggregated nanostructures, but also hold significant promise in various fields. In nanomedicine, for instance, the optical blaster could revolutionize targeted drug delivery, enabling the controlled positioning and ultrafast release of therapeutic agents at specific locations within cells and tissues.^4^ An intriguing possibility involves the transportation and launch of living cells attached to a CLC microcargo, offering a novel method for precise delivery to required tissue sites in microsurgery or for in vitro fertilization.^46,47^ The concept that we demonstrated on the microscale even has potential applications on the macroscale, particularly in launching and controlling the speed of a light-sailing spacecraft based on CLC materials.^8,9^ For instance, a CLC-based rocket could be launched by altering the handedness of the circularly polarized light from the driving laser source, whereas the speed of the spacecraft can be finely tuned not only by adjusting the laser intensity but also by changing the ellipticity of the polarization. This adaptation demonstrates the versatility of the optical blaster concept across various domains, showcasing its potential for advancing technology and scientific exploration.

## Methods

### Preparation and Characterization of CLC Polymer Particles

The CLC polymer particles were prepared by suspension polymerization under UV light irradiation according to our previous reports.^35,36^ Two types of CLC particles with planar and homeotropic alignments were successfully obtained using different surfactants, anionic sodium dodecyl sulfate^36^ (SDS, Sigma-Aldrich), and nonionic polyvinylpyrrolidone^35^ (PVP, Sigma-Aldrich), respectively. The preparation procedures for the homeotropic-alignment particles are as follows: 86.4 mg of 4-methoxyphenyl 4-((6-(acryloyloxy)­hexyl)­oxy) benzoate (RM105, Merck, Extended data Figure S5a), 86.4 mg of 1,4-Bis­[4-(3-acryloyloxypropoxy)­benzoyloxy]-2-methylbenzene (RM257, Merck, Extended data Figure S5b), 14.1 mg of right-handed chiral dopant 4′-[(*S*)-2-methylbutyl]­biphenyl-4-carbonitrile (CB-15, Merck, Extended data Figure S5c), 2.2 mg of photoinitiator Irgacure 819 (CIBA Inc.), and 1.2 mg of thermal inhibitor 2,6-di*tert*-butyl-4-methylphenol (Sigma-Aldrich) were dissolved in dichloromethane. The mixture was sonicated for 5 min to ensure homogeneity, and then the solvent was evaporated at 80 °C. In another flask, 200 mg of SDS as an ionic surfactant was dissolved in 20 mL of distilled water, which was then heated to 80 °C. This aqueous solution was poured into the monomer mixture at 80 °C, and the two-phase mixture was vigorously stirred using an IKA T18 digital ULTRA TURRAX homogenizer equipped with an IKA S18N-10G shaft generator at 15,000 rpm for 15 min. The emulsion formed was slowly cooled to ambient temperature to induce homeotropic alignment and then poured into a Petri dish. Subsequently, the Petri dish was placed inside a box flowing with nitrogen gas, and the emulsion was stirred on a magnetic stirrer at 300 rpm. The polymerization was carried out under UV-light irradiation at a maximum power of a mercury lamp (EXFO Omnicure S2000, λ = 350–450 nm) for 30 min. The particles were collected using an Eppendorf MiniSpin plus centrifuge. After the first centrifugation cycle with ethanol, the CLC particles were washed with tetrahydrofuran twice and dried under reduced pressure. The planar alignment CLC particles were also similarly prepared using 14.3 mg of PVP as a surfactant instead of SDS. The alignment of the cholesteric layers in the polymer particles (Figure 1a and 11b) was examined using transmission electron microscopy (TEM). This analysis was conducted with a Titan by FEI, which operates with a LaB6 filament at 300 kV under slight under-focus conditions. The particles were embedded in EPOFIX epoxy resin. Cross sections were prepared at room temperature using an ultramicrotome (Reichert-Jung Ultracut E) set to a thickness of 60 nm. The resulting sections were then transferred to a grid covered with a carbon film (Electron Microscopy Sciences, CF200-CU) for TEM analysis. For the optical trapping experiments, the dried powder of microparticles was resuspended in dodecane (Sigma-Aldrich) with 0.5 wt % of an OLOA surfactant (Merck) to prevent their agglomeration and sticking to the glass surface of the microfluidic chamber during the experiments.

### Analytical Description of the CLC Director Configuration

The full derivation of the analytical framework can be found in our previous reports.^15,32^ In short, the orientation of the director vector *L* for each *x*, *y*, and *z* coordinate is determined by the twist φ and tilt θ angles. For the planar CLC structure (Figure 1a), the director remains perpendicular to the helical axis, and the CLC material has a helical pitch *p*. The azimuthal angle of the director φ_p_ depends only on the *z*-coordinate and is given by φ_p_ = 2π*z*/*p*, with θ_p_ = π/2. Using these angles, the director components *L*_*x*_, *L*_*y*_, and *L*_*z*_ are expressed as^15^ (Figure 1c, top row):

1 $$L_{p}=\left(\begin{array}{l} L_{x}\\ L_{y}\\ L_{z} \end{array}\right)=\left(\begin{array}{l} \cos\;\varphi_{p}\;\sin\theta_{p}\\ \sin\varphi_{p}\;\sin{\;\theta }_{p}\\ \cos\theta_{p} \end{array}\right)$$

For particles with an onion-like structure of CLC layers (Figure 1b), we describe the director components *L*_*x*_, *L*_*y*_, and *L*_*z*_ (Figure 1c, bottom row) as a function of the spherical coordinates of the position: $r=\sqrt{x^{2}+y^{2}+z^{2}}$ , $\theta_{o}=\sqrt{x^{2}+y^{2}}/z$ , and φ_o_ = tan^−1^(*y*/*x*) with the following formula:

2 $$L_{o}=\left(\begin{array}{l} L_{x}\\ L_{y}\\ L_{z} \end{array}\right)=\left(\begin{array}{l} \cos{\;\theta }_{o}\cos{\;\varphi }_{o}\cos\left(\varphi_{o}+2\pi r/p\right)-\sin{\;\varphi }_{o}\sin\left(\varphi_{o}+2\pi r/p\right)\\ \cos{\;\theta }_{o}\sin\;\varphi_{o}\cos\left(\varphi_{o}+2\pi r/p\right)+\cos\;\varphi_{o}\sin\left(\varphi_{o}+2\pi r/p\right)\\ -\sin\;\theta_{o}\cos\left(\varphi_{o}+2\pi r/p\right) \end{array}\right)$$

The example in Figure 1c shows the particles with a diameter *d*_p_ of 6 μm and helical pitch *p* size of 600 nm. Note that the director on the positive *z*-axis depends on the value of φ_o_.

### Numerical Calculations of Optical Forces and Torques

Electromagnetic field simulations were conducted using COMSOL Multiphysics 5.6 (Wave Optics module) with a finite-element solver as previously reported.^30,32,36,48^ The model geometry contains a spherical particle with diameter *d*_p_ = 2 μm and chiral pitch *p* = 600 nm, immersed in dodecane, which serves as the surrounding medium (refractive index *n*_m_ = 1.42) and has a relative dielectric constant ε_*m*_ = *n*_m_^2^. Using the director components *L*_p_ and *L*_o_ (Figure 1c) according to eqs 1 and 2, we describe the optical properties of the particle with the relative dielectric tensor components ε_*ij*_:

3 $$\hat{\varepsilon_{ij}\;}=\varepsilon_{o}\delta_{ij}+\left(\varepsilon_{e}-\varepsilon_{o}\right)L_{i}L_{j}$$

where δ_*ij*_ is the Kronecker delta, and ε_o_ = *n*_o_^2^ and ε_e_ = *n*_e_^2^ are the ordinary and extraordinary relative dielectric constants, respectively. The refractive indices of the liquid crystal are *n*_o_ = 1.5 and *n*_e_ = 1.7 for free-space wavelength λ_0_ = 975 nm. The entire model is enclosed in a perfectly matched layer (PML) to absorb reflected and backscattered radiation from the model boundaries. The model is meshed with tetrahedral elements with a maximum element size of λ_0_/(5*n*_m_). The background electric field is modeled as a highly focused (numerical aperture NA 1.3) Gaussian beam^11,49^ with LCP and RCP. For each particle axial position (Figure 2), the electromagnetic field distribution is computed, and the resulting Maxwell’s stress tensor^11^ is determined as

4 $${\mathrm{T̂}}_{ij}=E_{i}D_{j}+H_{i}B_{j}-\frac{1}{2}\left(\mathrm{E⃗}\cdot \mathrm{D⃗}+\mathrm{H⃗}\cdot \mathrm{B⃗}\right)\delta_{ij}$$

where *E⃗*, *D⃗*, *H⃗*, and *B⃗* are the electric field, electric displacement, magnetic field, and magnetic induction vectors, respectively. By integrating this tensor over the enclosing surface *S* around the particle geometry, we calculate the time-averaged optical force^11^ (Figure 2, Extended data Figures S1a and S2):

5 $$\left\langle \mathrm{F⃗}\right\rangle =\oint_{S}^{.}\left\langle \mathrm{T̂}\left(\mathrm{r⃗},t\right)\right\rangle dS$$

and optical torque^11^ (Extended data Figure S1b):

6 $$\left\langle \mathrm{\tau ⃗}\right\rangle =-\oint_{S}^{.}\left\langle \mathrm{T̂}\left(\mathrm{r⃗},t\right)\times \mathrm{r⃗}\right\rangle dS$$

The calculated optical forces and torque are normalized by the power, *P*_0_, of the incoming light.

### Optical Trapping Experiments and Data Analysis

The optical trapping experiments were conducted using an optical tweezers setup^32,44^ based on an inverted optical microscope (Eclipse Ti, Nikon). A continuous-wave laser diode (λ_0_ = 975 nm, ITC4005, Thorlabs) was expanded and guided to an oil-immersion objective lens (100×, NA = 1.3, Plan Fluor, Nikon) to create the optical trap. To manipulate the circular polarization state for particle trapping and launching, we utilized a combination of a half-wave plate (HWP; WPH10M-980, Thorlabs), a liquid crystal (LC, E7, Merck) cell, and a quarter-wave plate (QWP; WPQ10M-980, Thorlabs), as shown in Extended data Figure S3. The laser power was set to 140 mW and measured at the entrance of the objective lens. The particle position was recorded using a CCD camera (iXon+, Andor) with a frame rate of up to 40 Hz. We measured the particle’s axial position and launching speed using diffraction pattern analysis.^50,51^ An area of interest (20 μm × 20 μm) around the particle was defined to isolate the single bead within the camera image (Figures 3a and 4). A lookup table was created before the experiment by capturing the diffraction pattern of the particle on the glass surface at different objective positions along the *z*-axis (Extended data Figure S4a), with the objective displaced using a moving stage in 100 nm steps. The particle radial (*x*, *y*) position was estimated using centroid detection.^52^ The diffraction pattern of the trapped and launched bead (the same one used for the lookup table) at any given video frame was compared to the lookup table (Extended data Figure S4b) using a cross-correlation algorithm to determine the axial (*z*) position as a function of time (Figure 3b). The extracted position data was used to estimate the launching speed by making a linear fit for the first 200 ms after the launch (Figure 3b). To measure the downward drag force, we apply Stokes’ law:

7 $$F_{drag}=3\pi \eta d_{p}v$$

with the viscosity η of the surrounding medium and upward shooting velocity *v*. To estimate the gravitational forces acting on the particle, we use Archimedes’ principle:

8 $$F_{grav}=\left(4/3\right)\pi R_{p}^{3}\left(\rho_{p}-\rho_{m}\right)g$$

where *R*_p_ is a particle radius, *g* is a gravitational constant, ρ_m_ and ρ_p_ are material densities of the surrounding medium and particle, respectively. The resulting optical force is estimated from balancing forces according to Newton’s third law of motion:

9 $$F_{opt}=F_{grav}+F_{drag}$$

## Supplementary Material



















## Notes

Sequence of video frames showing a microparticle with concentric CLC layers when the circular polarization of the trapping laser is switched (MOV)
The same behavior observed for the microparticles with the planar stacking of CLCs (MOV)
Particles with a planar stacking of CLC layers do not always maintain a trappable orientation after a second trapping attempt is made after releasing the particle (MOV)
Scattering force under RCP does not launch the particle with a small size (MOV)
Time-lapse images of CLC particle settling on the glass after the laser is switched off (MOV)
Same particle remains levitated above the laser focus for an extended period (MOV)
Intriguing effect when a couple of attached particles with similar sizes are loaded and launched out of the laser focus (MOV)
Juggling of three beads by loading and shooting them out of the optical trap for two juggling rounds (MOV)
Extended calculations of the optical forces and torques exerted on CLC microparticles; calculated optical forces acting on CLC microparticles with different helical pitches; schematic representation of the experimental setup; image data acquired for particle localization analysis; chemical structures of monomers applied for the synthesis of CLC particles (PDF)
