# Supplementary material for: Optical Blaster: Launching Nanostructured Microrockets out of an Optical Trap by a Single Laser Beam
Source: ACS Nano. 2025 Jul 18;19(31):28460–8. doi: 10.1021/acsnano.5c07197 (PMC12356119; doi:10.1021/acsnano.5c07197)
Supplement: Supplementary file 9 [file nn5c07197_si_009.pdf]

Optical Blaster: Launching Nanostructured Microrockets out of an Optical Trap by a Single Laser Beam

Yera Ussembayev[1,2†], Yuki Arakawa[3,4†], Filip Beunis[1,2], Anne B. Spoelstra[3,5], Tom Bus[3], Albert P. H. J. Schenning[3], and Kristiaan Neyts[1,2,6]**

[1]LCP research group, Ghent University, Technologiepark 126, 9052 Gent, Belgium
[2]Center for Nano and Biophotonics, Ghent University, Technologiepark 126, 9052 Gent, Belgium
[3]SFD research group, Eindhoven University of Technology, 5600 MB Eindhoven, The Netherlands
[4]Department of Applied Chemistry and Life Science, Toyohashi University of Technology, Toyohashi 441-8580, Japan
[5]CMEM research group, Eindhoven University of Technology, 5600 MB Eindhoven, The Netherlands
[6]Hong Kong University of Science and Technology, Clear Water Bay, Kowloon, 000000, Hong Kong

** corresponding authors: a.p.h.j.schenning@tue.nl and kristiaan.neyts@ugent.be*
† equally contributing authors

Supplementary Information

Number of pages: 3
Number of figures: 5

Content:

1. **Figures:**
   1.1 Figure S1. Extended calculations of the optical forces and torques exerted on CLC microparticles
   1.2 Figure S2. Calculated optical forces acting on CLC microparticles with different helical pitches
   1.3 Figure S3. Experimental setup
   1.4 Figure S4. Image data acquired for particle localization analysis
   1.5 Figure S5. Chemical structures of monomers applied for the synthesis of CLC particles

Supplementary figures

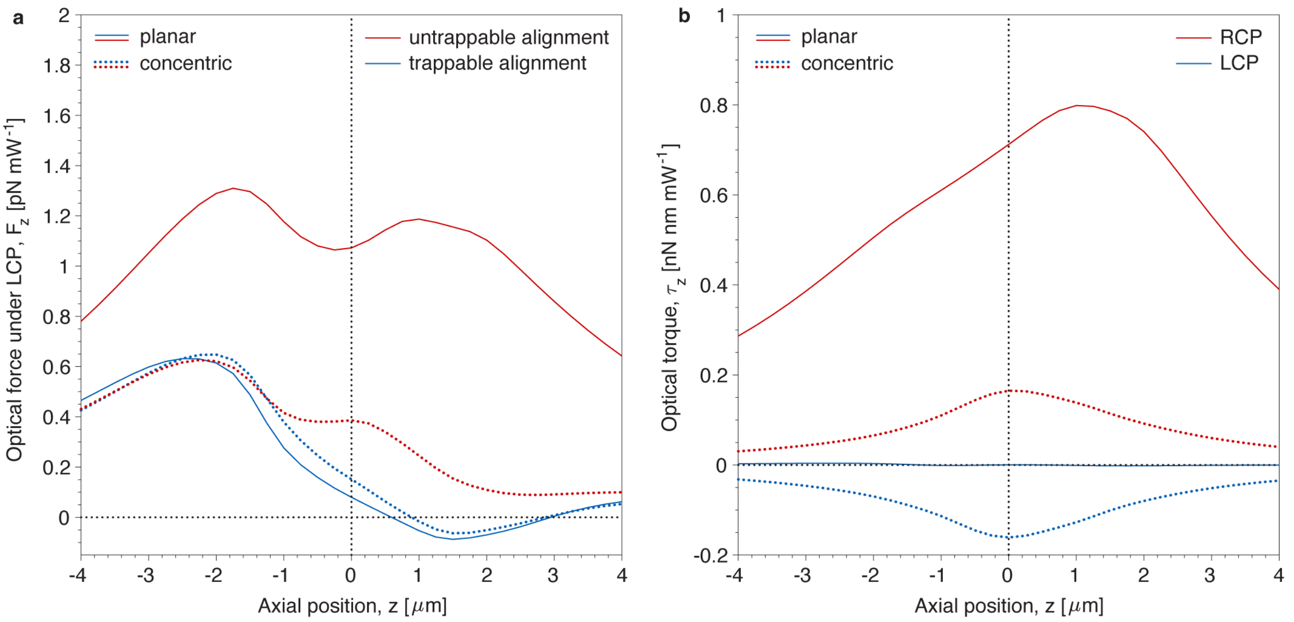

Figure S1. Extended calculations of the optical forces and torques exerted on CLC microparticles. a. Optical forces as a function of axial position under LCP. The blue lines respectively represent the force for planar particles with the helical axis parallel to the light propagation direction (solid) and concentric particles with the disclination line perpendicular to the light propagation direction (dotted), both resulting in an equilibrium position and stable optical trapping. The red lines respectively indicate the force acting on the particles with planar stacking aligned with the helical axis perpendicular to the propagation direction (solid) and onion-like particles with the helical axis parallel to the propagation direction (dotted), making them untrappable. **b**. Optical torques as a function of position, generated by LCP and RCP for the

beads with a planar stacking oriented with the helical axis parallel to the propagation direction and concentric particles with a defect line perpendicular to the light propagation direction.

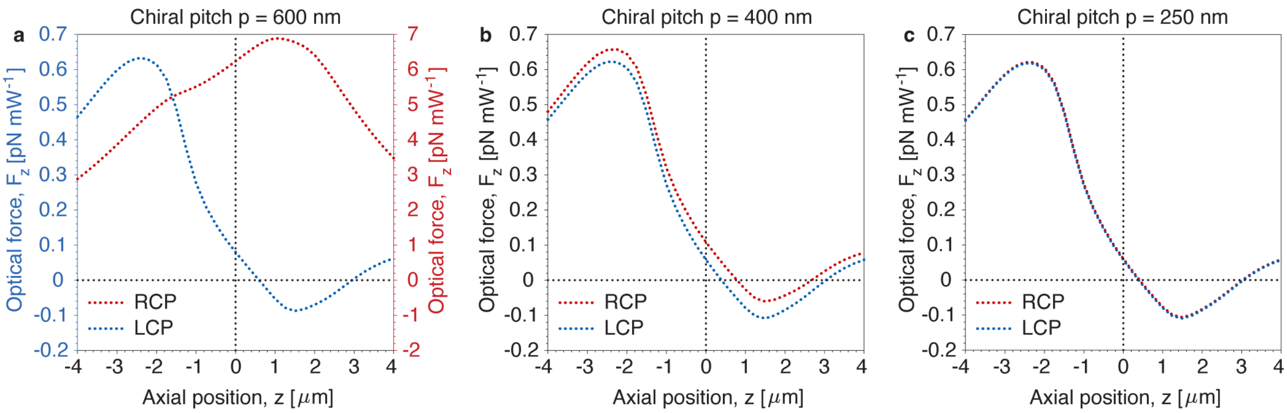

Figure S2. Calculated optical forces acting on CLC microparticles with different helical pitches. Optical forces as a function of axial position for LCP and RCP illuminating particles with planar stacking of CLC layers aligned with a helical axis parallel to the light propagation direction (helical axis along z) and helical pitches of 600 nm (**a**), 400 nm (**b**), and 250 nm (**c**), which form photonic bandgaps in the near-infrared, red, and blue wavelength ranges, respectively.

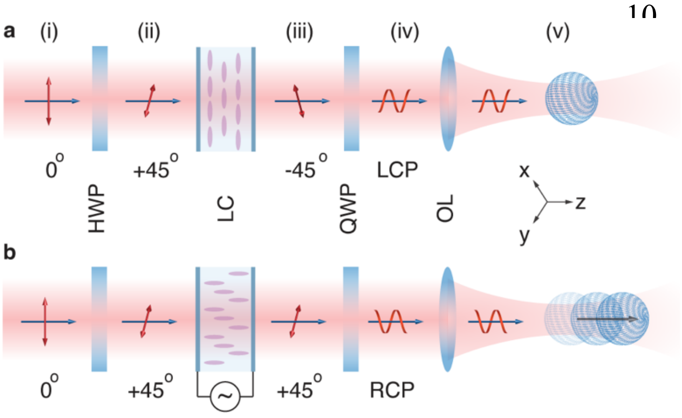

Figure S3. Experimental setup. a. Schematic illustration of the experimental setup, where the expanded NIR laser beam is guided through a half-wave plate (HWP), liquid crystal (LC) cell, quarter-wave plate (QWP), and a high NA objective lens (OL) to create an optical trap with left-handed circular polarization. **b**. Launching of the CLC particle by switching the circular polarization from left-handed (LCP) to right-handed (RCP) based on the applied AC voltage on the LC cell. Blue arrows indicate the wavevector, and red arrows/helices show the polarization states (i)-(iv) of the light after passing through different optical components.

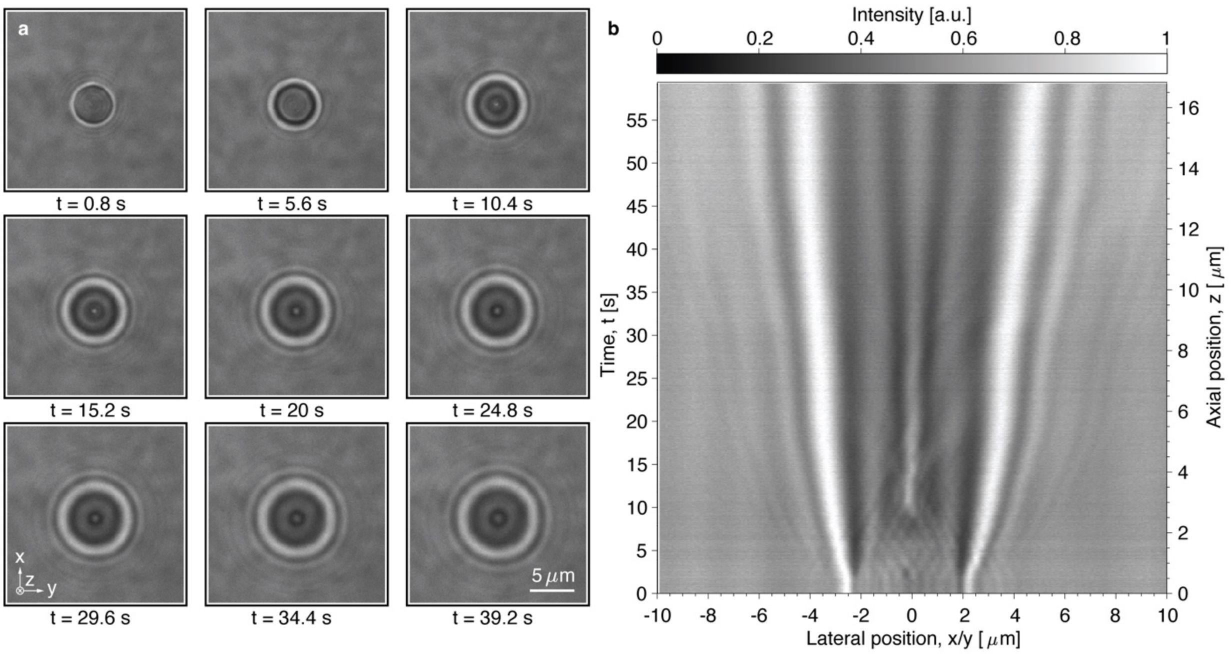

Figure S4. Image data acquired for particle localization analysis. a. Diffraction pattern of a CLC microparticle on a glass surface at different distances from the particle to the focal plane of the objective lens along the *z*-axis. **b**. Lookup table created from radial intensity profiles from (**a**) centered on the diffraction pattern images, captured at focal plane positions with 100 nm step intervals.

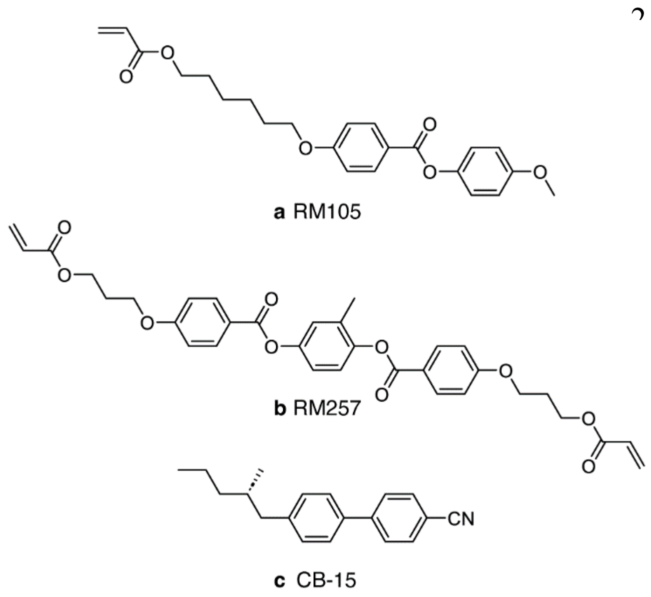

Figure S5. Chemical structures of monomers applied for the synthesis of CLC particles. **a.** 4-Methoxyphenyl 4-((6-(acryloyloxy)hexyl)oxy) benzoate (RM105). **b.** 2-Methyl-1,4-phenylene bis(4-(3-(acryloyloxy)propoxy)benzoate) (RM257). **c.** Right-handed chiral dopant (S)-4'-(2-Methylbutyl)-[1,1'-biphenyl]-4-carbonitrile (CB-15).
